# Community-based treatment of cutaneous leishmaniasis using cryotherapy and miltefosine in Southwest Ethiopia: the way forward?

**DOI:** 10.3389/fmed.2023.1196063

**Published:** 2023-10-10

**Authors:** Saskia van Henten, Myrthe Pareyn, Dagimawie Tadesse, Mekidim Kassa, Mehret Techane, Eyerusalem Kinfe, Nigatu Girma, Degnet Demeke, Mebratu Mesay, Mekibib Kassa, Rodas Temesgen, Misgun Shewangizaw, Fekadu Massebo, Johan van Griensven, Teklu Wegayehu, Behailu Merdekios

**Affiliations:** ^1^Department of Clinical Sciences, Institute of Tropical Medicine Antwerp, Antwerp, Belgium; ^2^Department of Medical Laboratory Sciences, College of Medicine and Health Sciences, Arba Minch University, Arba Minch, Ethiopia; ^3^Department of Public Health, College of Medicine and Health Sciences, Arba Minch University, Arba Minch, Ethiopia; ^4^Department of Dermatology, College of Medicine and Health Sciences, Arba Minch University, Arba Minch, Ethiopia; ^5^Department of Biology, College of Natural and Computational Sciences, Arba Minch University, Arba Minch, Ethiopia; ^6^Leishmaniasis Research and Treatment Center, University of Gondar Hospital, Gondar, Ethiopia; ^7^Department of Internal Medicine, College of Medicine and Health Sciences, Arba Minch University, Arba Minch, Ethiopia

**Keywords:** decentralization, patient-reported outcomes, impavido, treatment outcomes, operational research

## Abstract

**Background:**

Cutaneous leishmaniasis (CL) is a common, yet massively underreported skin morbidity in Ethiopia. Most patients never seek treatment, as this is offered only in specialized treatment centers. Early diagnosis and treatment through decentralization is crucial to decrease transmission and to reach the NTD roadmap goals. However, little information is available on outcomes and challenges of community-based treatment initiatives.

**Methods:**

A community-based prospective cohort study was conducted in Ochollo. Patients with clinically or microscopy confirmed CL were included. Cryotherapy was (to be) given weekly with at least four sessions for uncomplicated lesions, and miltefosine was given for 4 weeks for complicated lesions. Miltefosine adherence was assessed by counting pill strips. Clinical and patient-reported outcomes (dermatological life quality index and patient-global assessment) were assessed at month 6 (M6).

**Results:**

A total of 107 patients were included, with a median age of 6 years. Two patients refused, and 15 could not be treated as they were too young (<4 years) for miltefosine. Giving cryotherapy to patients weekly was not feasible due to long wound healing times and required use of topical antibiotics. Only 52.4% of miltefosine patients finished >90% of their tablets by M1. Among 46 patients treated with cryotherapy, 24 (52.2%) were cured at M6, and 9 (19.6%) had substantial improvement. The cure rate was 16/39 (41.0%) for miltefosine with 28.2% (11/39) substantial improvement. Before treatment, more than half (57.8%) of patients reported that CL did not negatively impact their life, which significantly increased to 95.2% at M6. At this time, 61.7% of patients said their lesion was clear, which was 1% before treatment.

**Conclusion:**

Our study is the first to identify the challenges and opportunities of miltefosine and cryotherapy for community treatment of CL. Although overall cure rates were lower than expected, patient-reported outcomes were generally positive and quite some patients had good improvement.

## Introduction

Cutaneous leishmaniasis (CL) is a neglected tropical disease (NTD) caused by *Leishmania* protozoa. In Ethiopia, the majority of the CL cases is caused by *Leishmania aethiopica*, with lesions generally severe, of long-standing duration and hard to treat compared to CL caused by other species (1).

Transmission of CL in Ethiopia is typically described to be zoonotic with hyraxes as its reservoir. However, studies by Mutinga et al. (2) and Pareyn and Kochora et al. (3) demonstrate that besides hyraxes, humans also seem to be an important reservoir that can fuel transmission of *L. aethiopica*. Therefore, early diagnosis and treatment of CL patients to tackle the human source of infection could be pivotal to decrease the disease burden (4).

Although the estimated yearly incidence of CL is 20,000 to 50,000, only 878 cases were reported to the WHO in 2018 (5, 6). These numbers show that there is severe underreporting of CL. This is primarily because CL diagnosis and care are only available in specialized treatment centers, often far from patients, impeding them to seek modern treatment. Decentralizing treatment closer to patients therefore seems crucial to obtain the NTD roadmap goal of detecting 85% of all cases and making sure 95% of them are treated (7).

However, the most widely available treatment, intramuscular or intralesional injections with sodium stibogluconate (SSG), is challenging to use in primary healthcare facilities. Cryotherapy (for smaller uncomplicated lesions) and miltefosine (for severe, complicated lesions) seem more suitable alternatives. Cryotherapy is used for the treatment of CL in Ethiopian referral hospitals (8, 9), although reports on its long term cure rate are scarce. It can be performed on an out-patient basis, has few side-effects, and is relatively cheap (10). Although liquid nitrogen comes with logistic challenges, projects in other countries have shown that administration of liquid nitrogen by nurses during field visits is possible, acceptable, and safe (11).

Miltefosine is the only available oral leishmaniasis treatment and is relatively safe with mostly mild gastro-intestinal adverse effects. Therefore, it has a good potential for outpatient treatment of CL patients who need systemic treatment. In a hospital-based study conducted in Northern Ethiopia, CL patients with severe, large, and long-standing lesions were treated with miltefosine in which it was found to be acceptable and safe. However, the effectiveness differed greatly between the two study sites (72.7% vs. 26.7% cure) (12). As we hypothesized that lesions in the community are more recent and less severe, community-based detection and earlier treatment of CL cases could improve the outcome of miltefosine treatment.

Most studies on CL treatment only consider clinical outcomes, while including patients perspectives by incorporating scar and quality of life evaluation is recommended (13). In this pilot project, we determined clinical and patient-reported outcomes of community treatment for CL with cryotherapy and miltefosine and also describe the challenges and opportunities that were encountered.

## Methods

### Ethics statement

This study was approved by the ethical review committees of the Institute of Tropical Medicine in Antwerp (1,513/21), the University Hospital of Antwerp (21/27/275), and Arba Minch University (IRB/1122/2021). Written informed consent was obtained from all participants or from the guardian/parent of patients below the age of 18, with additional assent collected for patients aged 12–17 years. All patients specifically provided consent for taking and using their lesion photographs, provided they could not be identified.

### Setting

This study was carried out in Ochollo, a village in the southwest of Ethiopia, around 25 km north of Arba Minch. Ochollo is a rural, highly endemic, and well-known focus of CL. Previous research showed that 5.5% of the primary school children had active CL and 60% had scars due to a *Leishmania* infection (14). The village is inhabited by approximately 5,000 residents. Basic health service packages are provided to patients by health extension workers at the health post. More complicated medical cases are referred to Arba Minch General Hospital. CL treatment is not available at the health post or nearby district health center; rather, CL patients seek care from traditional healers living in Ochollo, who generally provide treatment for free.

### Design

**Figure 1:**
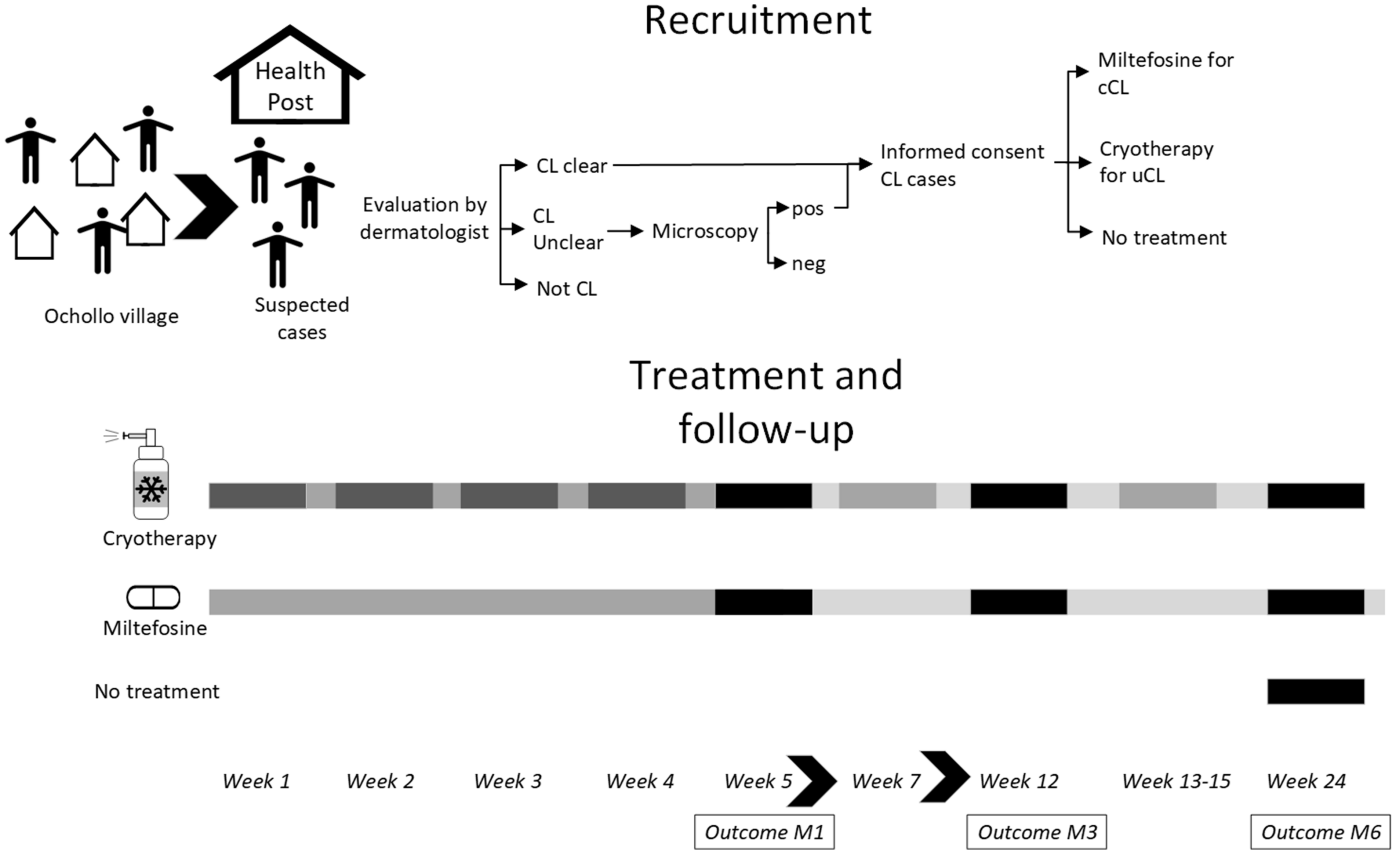
Study overview. All patients were enrolled in the first week of the study. Patients were screened by field assistants and asked to come to the health post. After evaluation by a dermatologists, patients were classified as clear CL, not CL or unclear CL, in which case a skin slit was taken for CL diagnosis by microscopy. Clear CL cases and those confirmed by microscopy were asked to give informed consent. Treatment allocation was done by a study physician: cryotherapy for uncomplicated CL (uCL) and miltefosine for complicated CL (cCL) patients. The next week, patients were started on treatment for four consecutive weeks, in which side-effects were also followed weekly. Treatment extension for cryotherapy could take place at week 5, 7, and 13–15 at the physicians’ decision. Outcome assessment (clinical and patient-reported) was done at month 1 (M1), month 3 (M3), and month 6 (M6) after starting treatment.

A prospective cohort study was conducted at the health post in Ochollo village from February until August 2022. Patients were treated with cryotherapy or miltefosine depending on the type of lesions with outcome assessment at 1, 3, and 6 months after starting treatment. The study procedures including recruitment, treatment, and follow-up visits at month 1 (M1), M3 and M6 are shown in Figure 1. This study follows the STROBE guidelines for reporting (15) (Supplementary material 1).

### Population and recruitment

Patients with suspected CL (as identified by four experienced field workers, self-identified, referred by the health extension worker, or identified by the study team at the market or school) were advised to visit the health post for formal evaluation during the first week of the study. A dermatologist or general practitioner with 3 years dermatology work experience evaluated the patients, and classified them as follows: (1) clear clinical diagnosis of CL, (2) clinically suspected CL, but lab confirmation needed, (3) not CL. Patients with clinical diagnosis of CL were immediately eligible for inclusion, while for patients whose clinical diagnosis was not sufficiently clear without lab confirmation, a skin slit for microscopy was collected. Any patients who were unwilling to give a non-invasive D-Squame Skin Stripping Disc (Monaderm, Monaco) sample (intended to confirm CL diagnosis by PCR) were excluded.

The dermatologist allocated the treatment to patients by first classifying patients as uncomplicated CL (<4 lesions and < 4 cm in size and no involvement of the nose, mucosa, or joints) or complicated CL (>4 lesions or lesion size >4 cm or involvement of mucosa, nose, joints, or signs of dissemination). Patients with uncomplicated CL were assigned to cryotherapy and patients with complicated CL to miltefosine. Complicated patients who were less than 4 years old could not be treated, as the protocol specified miltefosine could only be given to those above age four. Similarly, complicated patients who were pregnant or breastfeeding were not treated. Additionally, no treatment was given to patients who declined treatment, or for whom the physician did not deem treatment to be beneficial. These three patient groups who did not receive treatment were still enrolled for the study population description and outcome assessment at M6. No sample size calculation was done as we intended to recruit all voluntary and eligible patients from the village into the study.

### Sample collection and processing

A skin slit smear was collected from CL suspected patients for whom lab confirmation was needed by taking a sample with a scalpel from the border of the lesion. The skin slit was smeared on a microscopy slide, fixed with methanol, stained with Giemsa, and examined microscopically (1,000x magnification using oil immersion). Results were graded from negative to +6, based on WHO recommendations (16).

Two D-Squame Skin Stripping Discs were collected from every patient. The tape discs were placed one by one onto the border of the largest (index) lesion, pressed for approximately 10 s, and stored in 300 μL 1X DNA/RNA shield (Zymo Research, Baseclear, Netherlands) at −20°C.

An HIV test was performed for every patient using a fingerpick and HIV ½ STAT-PAK rapid test (Chembio diagnostic systems, inc., New York, United States).

For patients assigned to miltefosine treatment, blood was collected and checked the same day at Arba Minch General Hospital for creatinine, urea, AST, and ALT, as well as complete blood count. Results were discussed with an internist before patients were cleared for treatment. A urine dipstick pregnancy test was performed for every woman of child-bearing age and an intramuscular contraceptive was provided prior to and 3 months after starting miltefosine treatment.

### Molecular confirmation of cutaneous leishmaniasis

The first D-Squame Skin Stripping disc in 300 μL 1X DNA/RNA shield was subjected to extraction with the Maxwell 16 LEV Blood DNA kit (Promega, Netherlands) as specified in the manufacturer’s manual using the automated Maxwell 16 Instrument (AS1000, Promega). Nucleic acids were subsequently tested by qPCR targeting kinetoplast DNA (kDNA) as described by Merdekios et al. (17). The specific primers and probes used were LC-F (5’-TATTTTACACCAACCCCCAGT-3′), LC-R (5’-GGTAGGGGCGTTCTGC-3′) and a FAM-labeled LC-probe (5’-CAGAAAYCCCGTTCAAAAAATGGC-3′). If a sample was negative, the second D-Squame Skin Stripping disc was tested. If there was still no fluorescence, an HBB PCR was performed as described by Steinau et al. (18) to assess if there was sufficient tissue on the discs and whether the DNA extraction was conducted successfully.

### Lesion assessment

Lesion size, number, and type of lesion [localized CL (LCL), muco-cutaneous CL (MCL) or diffuse CL (DCL); (19) and detailed lesion characterization] were recorded at baseline. The largest lesion was classified as the index lesion. Photographs were taken at all timepoints to allow for comparison for outcome assessment and external cross-checking of lesion types, characterization, and outcomes.

### Patient reported outcomes and scar scale

Study staff administered the Dermatology Life Quality Index (DLQI) questionnaire to patients at baseline, M1, M3, and M6. For children up to 8, the questions were mainly asked to the parent/guardian, while for those aged 8–12, questions were asked both to the parent/guardian and the child. For children above 12, only the child’s answers were considered. Questionnaires were scored and analyzed as previously reported (20). Only questionnaires with 8 or more answered questions were analyzed, others were invalid (21).

Patient-reported outcomes were assessed by asking patients to rate the severity of their lesion at baseline, M1, M3, and M6, ranking it as clear, almost clear, mild, moderate, or severe. A modified Vancouver scar scale (mVSS) according to (22) was used to grade scar appearance at all timepoints after treatment.

### Treatment

Crusted lesions were first soaked with sterile saline to clean and remove the crust (if any). Cryotherapy was given using the CryoPro cryogun (Cortex Technology, Aalborg, Denmark), using at least 2 freeze–thaw cycles per application. Liquid nitrogen was applied on the lesion until the lesion and 1-2 mm margin of healthy skin was frozen, which took around 5–30 s, depending on the lesion size and thickness. The lesion was allowed to thaw, which took around 20–30 s, also depending on the freezing time and lesion size and thickness. After cryotherapy, patients were instructed to keep the lesion clean and all received 2% fusidic acid cream (Fusiderm, EVA-PHARMA, Egypt) to apply on the lesion daily. Cryotherapy was planned for 4 weeks with weekly application, but was withheld if lesions were still ulcerative, exudative, crusted, edematous or blistered after the previous application. Cryotherapy was extended if the physician considered it advantageous for the patient (Figure 1).

Miltefosine was given to patients to take at home, after instructing patients on their daily schedule, intake with food, and possible side-effects. Allometric dosing was used for children below 30 kg according to Dorlo et al. (23), 100 mg per day was given for patients of 30–44 kg, and 150 mg/day for patients of 45 kilos or above. Color-matched stickers were used on the pill strips and adherence monitoring forms to help patients take the correct dose in the morning and evening. For very young children, parents were advised to dissolve miltefosine in water mixed with sugar. Fusidic acid was given to patients allocated to miltefosine who had severe crusting or superinfection.

### Follow-up visits, safety, and adherence

Patients were asked to come every week during the first 4 weeks for treatment follow-up. Side-effects were recorded and graded according to the principles of the common terminology criteria for adverse events (24) (CTCAE). Adherence was monitored for miltefosine treatment. Every week, pill count was done based on the used pill strips, the daily missed doses were checked on the adherence form, and patients were asked how they took the medication. Poor adherence was defined when patients took less than 90% of their total dose at the M1 visit.

### Outcome assessment

Lesion outcomes were determined at M1, M3, and M6 based on the physician’s assessment. Patients were categorized as cured if all lesions present at baseline had shown 100% flattening and reepithelization in case lesions were ulcerated. Patients were considered substantially improved if all treated lesions had at least 50–99% improvement compared to baseline in terms of flattening (and reepithelization if applicable), while minor improvement required all lesions to have at least 1–49% improvement. Worsening was used if any treated lesions was worse than at baseline, whereas worsening at M6 compared to M3 was also recorded. New lesions were separately assessed.

### Data collection and analysis

Data was collected on paper-based forms, and double data entry was done in RedCap (25). Data analysis was done using R version 4.1.3 (26). Numbers, proportions, medians, and interquartile range (IQR) were used to describe the population. Treatment outcome was analyzed as a categorical variable with multinomial confidence intervals (CIs). Cure rate was also calculated as proportion (with 95% binomial CIs). Outcomes were calculated per treatment category. Subgroup analyses of treatment outcomes were done for outcomes using the index lesion only, by dosing class (allometric vs. non-allometric), adherence to miltefosine (poor adherence defined as <90% of total dose at M1), age group (<5 and > 5) and number of cryotherapy sessions. Treatment outcomes of these different groups were compared using chi-square tests, or Fisher-exact tests if chi-square tests were inaccurate. McNemar’s test was used to compare paired categorical data at different timepoints. DLQI-scores and patient-reported scores at different visits were compared with the Wilcoxon signed rank test for paired data, whereas the Mann–Whitney test was used to compared scores between different groups. Agreement between cure as assessed by the physician and whether the patient indicated the lesion to be clear was determined using kappa coefficient.

## Results

### Study population

A total of 147 patients were screened, of which 107 were included (Supplementary Figure 1). Of them, 95 (88.8%) were confirmed by PCR, three (2.8%) only by microscopy but not PCR, and nine (8.4%) clinically only. Forty-eight (44.9%) patients were allocated to cryotherapy, 42 (39.3%) to miltefosine, 15 (14.0%) could not be treated and two did not want to be treated. Thirteen of these patients could not be treated because they needed systemic treatment but were excluded from getting miltefosine as they were below 4 years old, one was pregnant, and for one the dermatologists decided not to treat.

**Figure 2:**
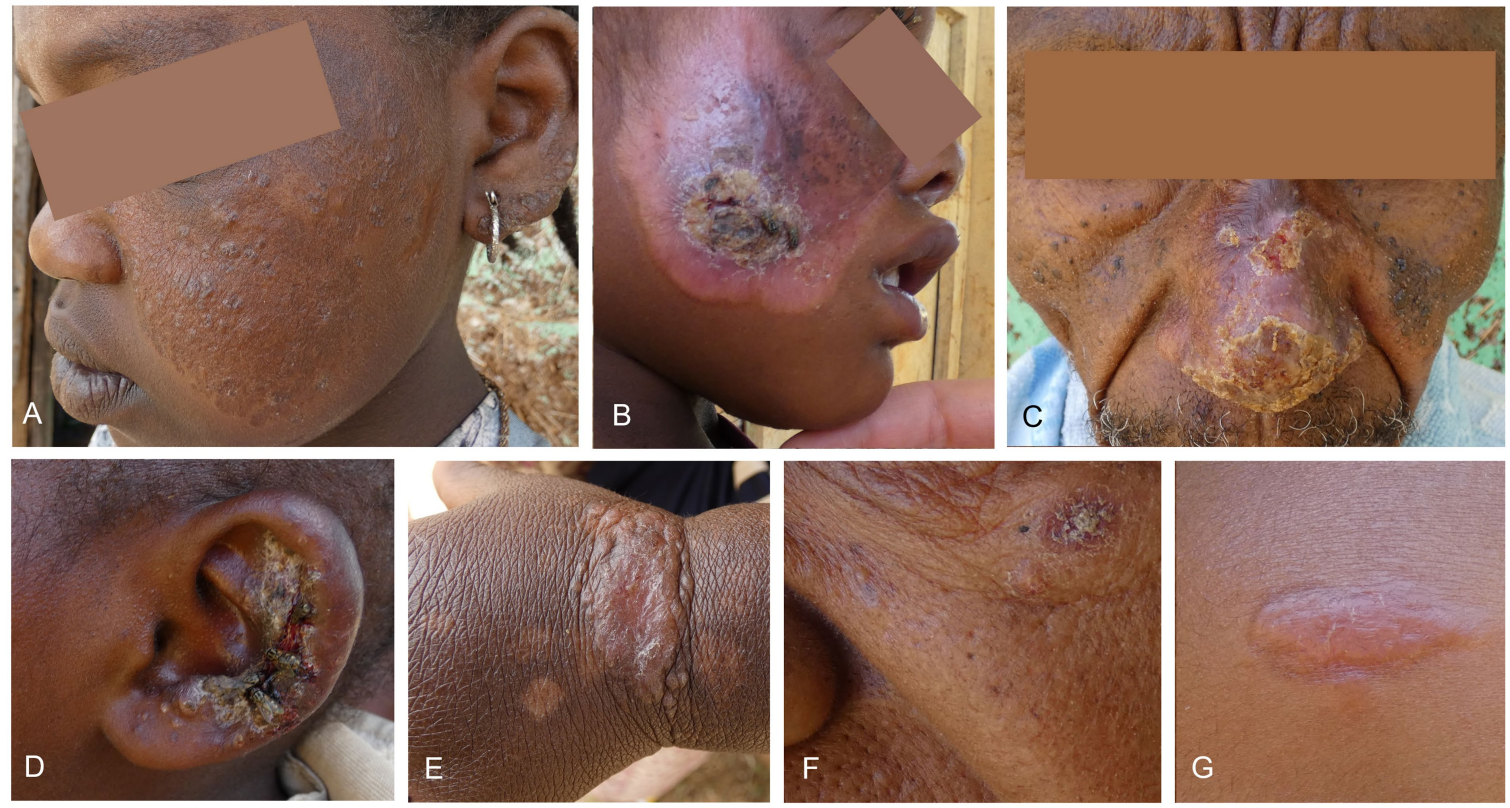
Lesion photographs of included patients. **(A)** An extensive lesion affecting the cheek and ear that the patient had as long as they can remember with active papules within a bigger scar, treated with miltefosine. **(B)** Superinfected lesion with scarring on the cheek, treated with miltefosine and systemic antibiotics. This patient had a contracture of the eye due to scar formation. **(C)** Crusted lesion on the nose treated with miltefosine. **(D)** Superinfected swollen lesion on the ear. No leishmaniasis treatment was given since the lesion was too big for cryotherapy and the patient was too young for miltefosine. Fusidic acid cream was given. **(E)** Plaque with papules on the wrist, treated with cryotherapy. **(F)** Small, crusted lesion underneath the eye treated with cryotherapy. **(G)** Nodular plaque lesion on the forehead treated with cryotherapy.

The study population is described in Table 1, with some photographs of included patients in Figure 2. Patients were young, with a median age of 6 years old. Only 13 (12.1%) patients were adults. About 60% of the patients were male. Almost half had used traditional (mostly herbal) treatment previously, which was 64% in the miltefosine group. Only 3 patients had used modern treatment at the hospital (probably SSG). A quarter of included patients had at least another CL case in the house, and almost three quarters (72.9%) reported having someone with a CL scar living in their home.

**Table 1:** Description of the study population.

Total	Total *N* = 107^a^	Cryotherapy *N* = 48 (44.9)	Miltefosine *N* = 42 (39.3)	No treatment *N* = 17 (15.9)
Age, median (years)	6.0 (3.0–11.0)	4.5 (2.0–10.0)	9.0 (6.0–12.0)	3.0 (1.5–3.0)
Sex, male	66 (61.7)	26 (54.2)	32 (76.2)	8 (47.1)
CL case in house	28 (26.2)	12 (25.0)	13 (31.0)	3 (17.6)
CL scar in house	78 (72.9)	34 (70.8)	30 (71.4)	14 (82.4)
Previous CL episode	17 (15.9)	11 (22.9)	5 (11.9)	1 (5.9)
Traditional treatment	53 (49.5)	14 (29.2)	27 (64.3)	12 (70.6)
Herbal^b^	27 (25.2)	3 (6.3)	16 (38.1)	8 (47.1)
Pressing	14 (13.1)	5 (10.4)	5 (11.9)	4 (23.5)
Burning	10 (9.3)	5 (10.4)	5 (11.9)	0 (0)
Other	3 (2.8)	1 (20.8)	2 (4.8)	0 (0)
Modern treatment^c^	7 (6.5)	1 (20.8)	6 (14.3)	0 (0)
Duration in months, median (IQR) (*n* = 106)	12.0 (6.0–24.0)	8.0 (4.0–24.0)	12.0 (12.0–45.0)	12.0 (4.0–12.0)
Type of lesion				
LCL	86 (80.4)	48 (100)	26 (61.9)	12 (70.6)
MCL	19 (17.8)	0 (0)	14 (33.3)	5 (29.4)
DCL	2 (1.9)	0 (0)	2 (0.5)	0 (0)
Number of active lesions				
1	50 (46.7)	34 (70.8)	11 (35.7)	5 (41.2)
2	17 (15.9)	6 (12.5)	7 (26.2)	4 (29.4)
3	13 (12.1)	3 (6.3)	9 (21.4)	1 (23.5)
≥4	27 (25.2)	5 (10.4)	15 (35.7)	7 (41.2)
Presence of concomitant CL scar	41 (38.3)	16 (33.3)	21 (50.0)	4 (23.5)
Size (largest diam), median IQR (*n* = 100)	2.2 (1.4–3.4)	1.6 (1.2–2.5)	3.5 (1.9–5.5)	2.1 (1.6–3.0)
Location index on face	97 (90.6)	44 (91.7)	37 (88.1)	16 (94.1)

CL, cutaneous leishmaniasis; DCL, diffuse cutaneous leishmaniasis; IQR, interquartile range; MCL, muco-cutaneous leishmaniasis; IQR, inter-quartile ranges. ^a^If a different denominator is used, this is indicated behind the variables as (*n* = x), ^b^For herbal medication, the traditional healers use different kinds of plant extracts ^c^three patients took anti-leishmania treatment at the hospital, four used non-specific modern treatment (two ointments, one disinfectant, one antibiotic injection).

The median lesion duration was around 1 year, but five patients (aged 1, 6, 8, 10 and 11), indicated that they had their lesions for their whole life. Most patients had a single lesion on the face, although more than a quarter had four or more lesions. Around 80% of patients were classified as LCL, 19 (17.8%) as MCL and only 2 (2.8%) as DCL. Overall, more than a third of patients had both an active lesion and concomitant scar. This was 50% among patients on miltefosine treatment, who also had slightly longer duration of their lesions. The most common lesion presentations were plaque (61/107, 57.0%), erythema (56/107, 52.3%), scaliness (49/107, 45.8%), and hyperpigmentation (40/107, 37.4%), whereas ulceration (8/107, 7.5%) and nodules (19/107, 17.8%) were uncommon. All patients were HIV negative.

For most patients, CL had only a minor impact on their life (Supplementary Table 1), with 37/64 (57.8%) with a valid DLQI questionnaire having a score indicating no effect, and 22/64 (34.4%) having a small effect. For two patients (3.1%), the DLQI score indicated CL had a very large effect on their life. DLQI scores were significantly higher in adults than in children (*p* = 0.021, Mann–Whitney test). There was no difference in DLQI scores between males and females (*p* = 0.601, Mann–Whitney test).

### Cryotherapy treatment: follow-up and side-effects

Patients assigned to cryotherapy received from 1 up to 10 sessions, with a median of 4.5 (IQR 3.0–7.0). Cryotherapy was usually given in 2 or 3 freeze–thaw cycles, with a freeze–thaw duration of 5–30 s, depending on the lesion thickness, size, and patient cooperation. For many young children, the application of cryotherapy was challenging, requiring multiple short freeze–thaw cycles. At M1, only 9/48 (18.8%) patients had received four cycles, as was planned in the protocol. Cryotherapy was often postponed as lesions were still healing from the previous application, and patients were given fusidic acid as topical antibiotic daily for open wounds. Twenty-five patients who were not yet cured at M1 were extended on cryotherapy treatment at week 4 (M1) up to week 6, and week 13 (M3) up to 17.

**Figure 3:**
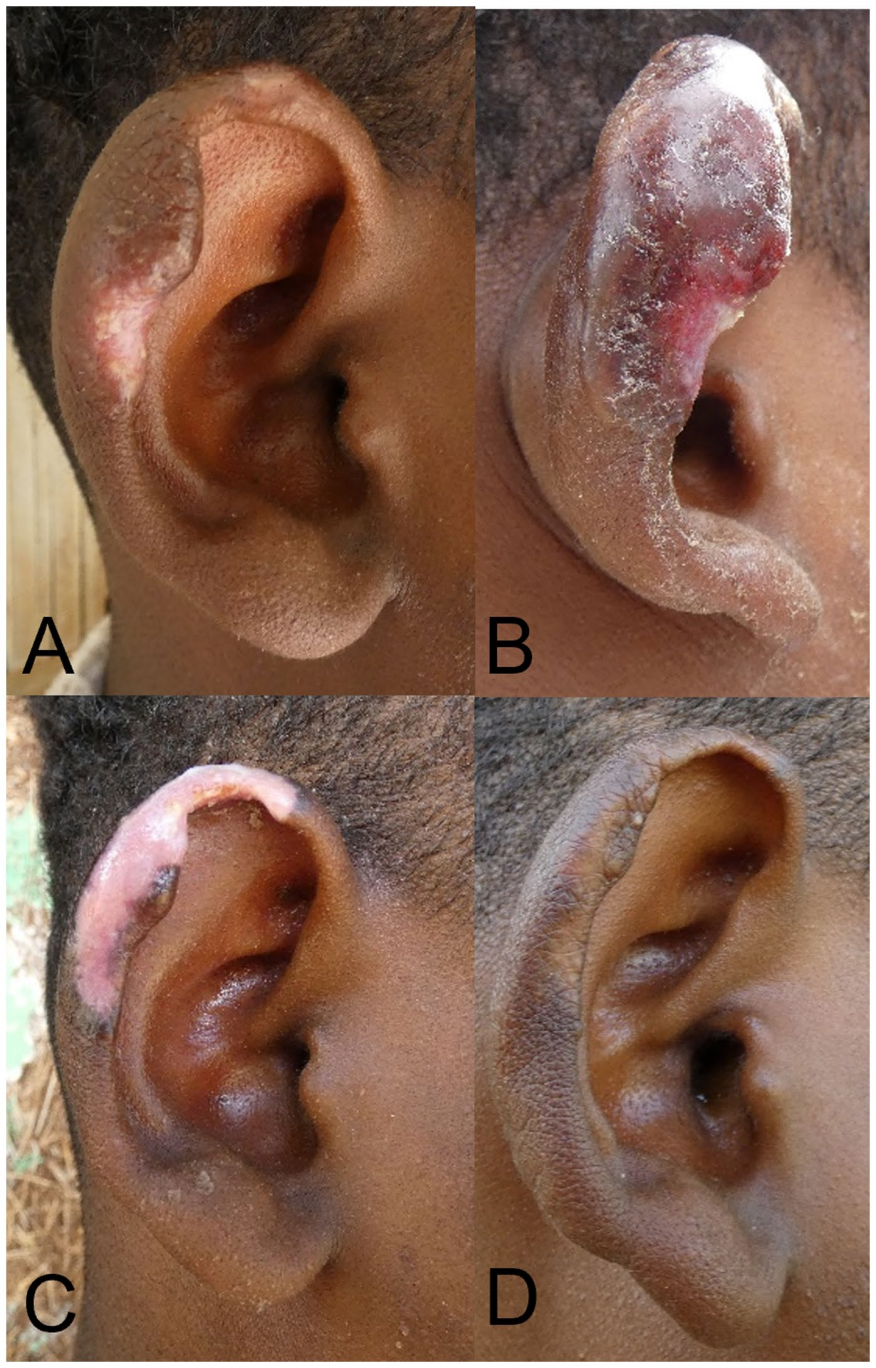
Patient with localized cutaneous leishmaniasis treated with cryotherapy. **(A)** Lesion before treatment. **(B)** Lesion 3 days post-cryotherapy with swelling and blistering. **(C)** Lesion at Month 1, with severe hypopigmentation. **(D)** Lesion at M6 with normal pigmentation and clinical cure.

Almost all patients treated with cryotherapy (43/48, 89.6%) experienced side-effects (Supplementary Table 2). Blistering (37/48, 77.1%), swelling (34/48, 70.8%) and infection (28/48, 58.3%) were common in the first week of treatment (Figure 3B), while pigmentation changes became more frequent after several cryotherapy applications (Figure 3C), but mostly recovered over time (Figure 3D). Most side-effects were mild (grade I), but several patients developed grade II events with infection (7/48, 14.6%) swelling (3/48, 6.3%) and blistering (4/48, 8.3%) for which three patients were given systemic antibiotics.

### Miltefosine treatment: adherence and side-effects

Generally, adherence forms were completed poorly despite repeated explanation. By counting the pill strips, compliance to miltefosine was irregular and poor. Eleven patients (26.2%) finished their complete course at M1 while 20 patients (47.6%) had poor adherence as they had taken less than 90% of their treatment and were advised to continue.

Side effects were common in patients treated with miltefosine (see Supplementary Table 3), with three quarters (76.2%) of patients experiencing adverse events. Most common were vomiting (20/42, 47.6%), abdominal pain (14/42, 33.3%), diarrhea (9/42, 21.4%) and nausea (14/42, 33.3%). Most side-effects were of severity grade I, a few of grade II, and one patient experienced severe bloody diarrhea with vomiting, abdominal pain, headache, and weakness, graded as severity III. The patient was treated with antibiotics for acute bacterial diarrhea and responded well, but had to discontinue her miltefosine treatment after 3 weeks.

### Treatment outcomes

Patient follow-up was good, with loss-to-follow-up below 10% in all treated patient groups at all visits (Supplementary Figure 1). Outcomes for cryotherapy are shown in Table 2. Around a quarter of the patients were cured after 1 month, which increased to 45.8% (95% CI 33.3–61.8) at M3 and 52.2% (95% CI 39.1–67.6) at M6. An additional 19.6% had substantial improvement at M6. Around 20% (21.7%) showed worsening at M6 compared to before treatment. Analyzing only the index lesion (Supplementary Table 4) did not affect the cure rates. Outcomes in children below 5 years old treated with cryotherapy were significantly different (*p* = 0.036, Fisher-exact test) to those aged 5 years and above (Supplementary Table 5). Although the overall cure rate was similar, young children more frequently had worsening lesions and less substantial improvement.

**Table 2:** Outcomes of cryotherapy for all lesions present at baseline.

	Month 1		Month 3		Month 6	
	*N* = 47 (97.9%)	95% CI	*N* = 48 (100%)	95% CI	*N* = 46 (95.8%)	95% CI
Cure	12 (25.5)	12.8–40.7	22 (45.8)	33.3–61.8	24 (52.2)	39.1–67.6
Substantial improvement	25 (53.2)	40.4–68.4	15 (31.3)	18.8–47.2	9 (19.6)	6.5–35.0
Minor improvement	6 (12.8)	0–28.0	4 (8.3)	0–24.3	2 (4.3)	0–19.8
No improvement	2 (4.3)	0–19.4	0 (0)	0–16.0	1 (2.2)	0–17.6
Worsening	2 (4.3)	0–19.4	7 (14.6)	2.1–30.6	10 (21.7)	8.7–37.2

**Figure 4:**
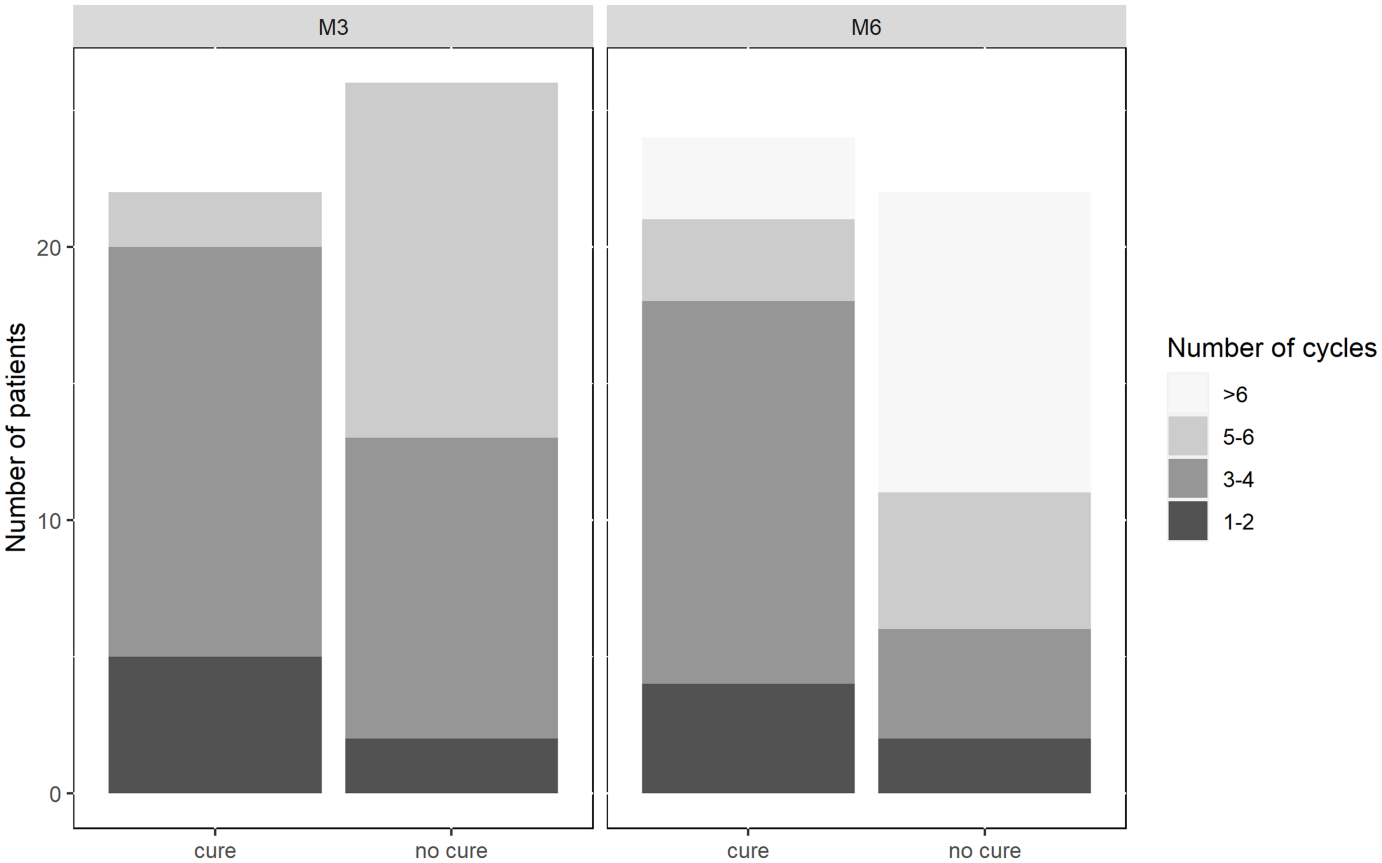
Cure rates of cryotherapy at Month 3 and Month 6 by number of cycles. Most patients (20/22) who were cured at M3 had received less than four cycles. At Month 6, three extra patients were cured with 5–6 cycles, and three who received more than six cycles. Around half (11/21) of the patients who were not cured at M6 had received more than six cycles of cryotherapy.

Despite extending treatment sessions up to 10 times, many patients receiving cryotherapy did not reach cure (Figure 4). In fact, data shows that cure rates are significantly lower for patients getting more than four sessions of cryotherapy (37.5% for 5–6 sessions and 21.4% for 7–8 sessions) compared to those who were treated with cryotherapy less than four times (*p* = 0.006 for M3 and *p* = 0.003 for M6, chi-square test).

Treatment outcomes for miltefosine are shown in Table 3. The overall cure rate for patients receiving miltefosine was 36.8% (95% CI 23.7–55.3) at M3 and 41.0% (95% CI 25.6–57.0) at M6. An additional 28.2% (95% CI 12.8–44.2) had substantial improvement at M6. In contrast, 20.5% (5.1–36.5) of the patients had lesions classified as worse than baseline at M6. Looking only at the index lesion outcomes (Supplementary Table 6), results are slightly better, with 51.3% of patients cured, 25.6% substantially improved, and only 10% worsening. There was no significant difference (*p* = 0.521, Fisher-exact test) in treatment outcomes in children receiving allometric dosing (weight below 30 kg) compared to children on non-allometric dosing (Supplementary Table 7). Outcomes were not significantly different for those with good adherence compared to those with poor adherence (*p* = 0.162, Fisher-exact test).

**Table 3:** Outcomes of miltefosine treatment for all lesions present at baseline.

	Month 1		Month 3		Month 6	
	*N* = 39 (92.9%)	95% CI	*N* = 38 (90.5%)	95% CI	*N* = 39 (92.9%)	95% CI
Cure	1 (2.6)	0–19.7	14 (36.8)	23.7–55.3	16 (41.0)	25.6–57.0
Substantial improvement	23 (59.0)	46.2–76.1	17 (44.7)	31.6–63.2	11 (28.2)	12.8–44.2
Minor improvement	15 (38.5)	25.6–55.6	4 (10.5)	0–28.9	3 (7.7)	0–23.7
No improvement	0 (0)	0–17.2	0 (0)	0–28.9	1 (2.6)	0–18.5
Worsening	0 (0)	0–17.2	3 (7.9)	0–26.3	8 (20.5)	5.1–36.5

Almost one fifth (18/100, 18.0%) of all patients developed a new lesion by M6, which was more common in patients getting local treatment with cryotherapy (10/46, 21.7%), and in those not treated (5/15, 33.3%). Three patients (3/39, 7.7%) on miltefosine treatment developed new lesions at M6. Additionally, almost a quarter of patients had worsening of their index lesion at M6 when compared to M3, which was especially pronounced in the group treated with miltefosine, where more than 30% (11/36, 30.6%) had worsening at M6 compared to M3.

Fifteen out of 17 patients who were not treated could be found at M6 for outcome assessment (Table 4). Of these, eight (53.3%) were cured, although 95% CI were very large (95% CI 33.3–79.8%). Two showed substantial improvement (13.3, 95% CI 0–39.8) and three patients (20.0, 95% CI 0–46.5%) were classified as worsening. Three of the untreated patients had reported the use of traditional herbal medication in between the initial assessment and the M6 outcome visit, of which two were cured. At least eight received topical fusidic acid at the start of the study, as the lesions looked crusted or infected. Of these, four were cured, two had substantial improvement, one minor improvement and one worsening.

**Table 4:** Outcome for patients not receiving treatment.

	Month 6	
	*N* = 15 (88.2%)	95% CI
Cure	8 (53.3)	33.3–79.8
Substantial improvement	2 (13.3)	0–39.8
Minor improvement	1 (6.7)	0–33.2
No improvement	1 (6.7)	0–33.2
Worsening	3 (20.0)	0–46.5

### Scar assessment

At M6, 79.0% (79/100) of patients had a remaining scar, which was similar for those who had received cryotherapy (36/46, 78.3%), miltefosine (32/39, 82.0%), or no treatment at all (11/15, 73.3%), and the median overall modified Vancouver scar scale was also similar for the different groups at 1.0 (Supplementary Table 8). Most scars (52/79, 65.8%) had slight pigmentation issues while a subset of patients had moderate (17.7%) or severe (2.5%) hypo- or hyperpigmentation. Although more than half the patients had normal pliability and height of scars, a subset had supple, yielding, or firm scars, and around 45% of the patients with scars had a scar that was raised.

### Patient reported outcomes

**Figure 5:**
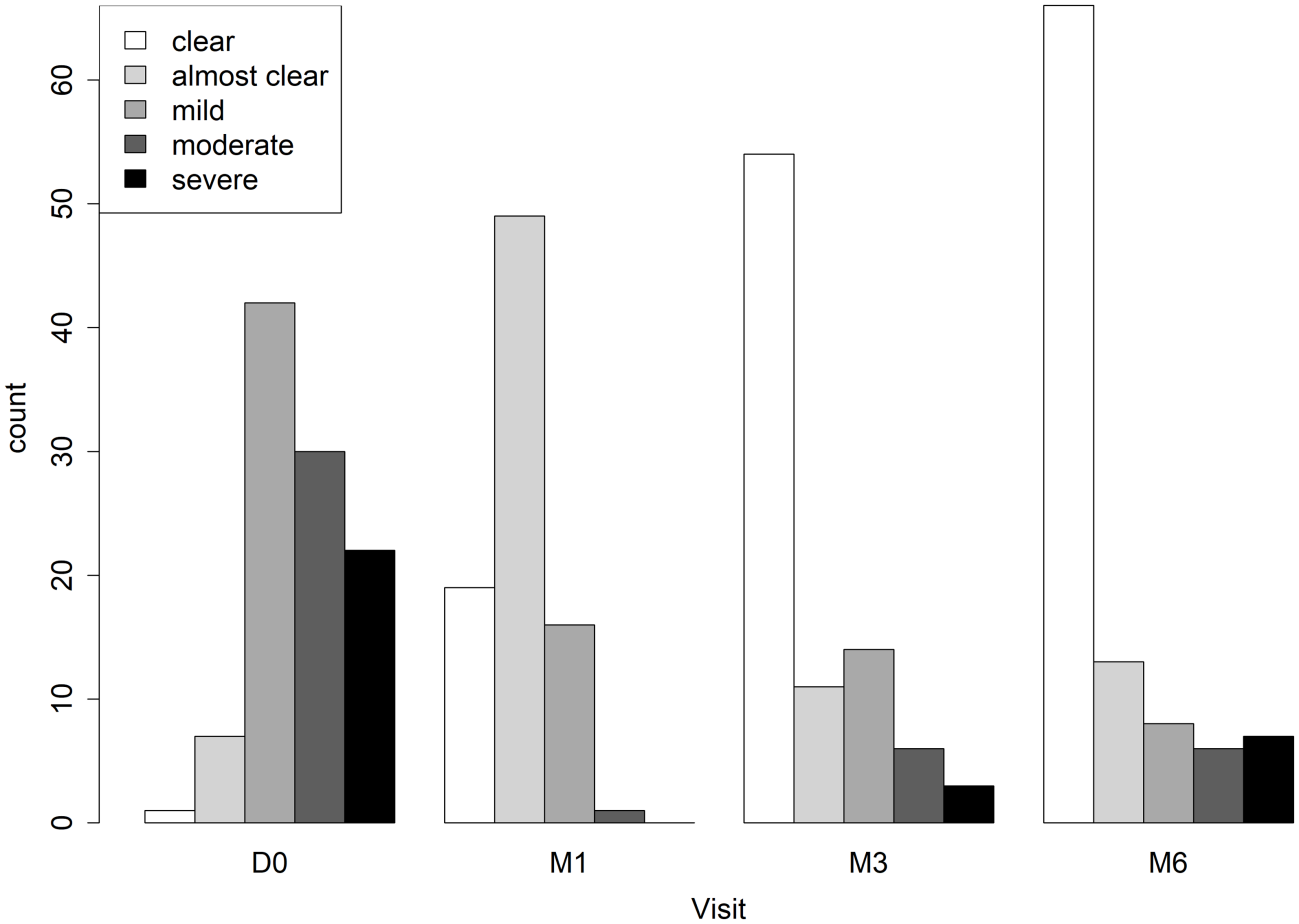
Patient reported lesion assessment at baseline and outcome visits. Patient-reported global assessment rated as clear, almost clear, mild, moderate, and severe is shown before treatment (D0), 1 month after starting treatment (M1), 3 months after starting treatment (M3), and 6 months after starting treatment (M6).

Patient reported outcomes over time are shown in Figure 5. Most patients rated their lesion as mild (39.3%), moderate (28.0%), or severe (20.6%) at the start of the study. Outcomes were significantly different at each follow-up visit (all *p* < 0.001, Wilcoxon Signed-rank -square test) compared to before treatment with 19 (17.8%) patients who said their lesion was cured at M1, 54 (50.5%) at M3, and 61.7% at M6.

DLQI scores significantly (*p* < 0.001, Wilcoxon Signed-rank Whitney test) changed over time with the median DLQI score rated 1 (IQR 0–3) before treatment and 0 (IQR 0-0)at M6. The proportion of patients who experienced negative effects on their quality of life due to their skin condition also decreased from 42.2% (27/64) before treatment to only 4.8% (3/63) at M6.

Overall, significantly less (*p* < 0.001, McNemar’s test) patients experienced pain in the lesion before treatment (37.7%, 40/106) compared to M6 (3.0%,3/100; Supplementary Table 9). The change was significant for cryotherapy (*p* < 0.001) and miltefosine (*p* = 0.004), but not for the patients not treated (*p* = 0.845,McNemar’s test). Similar results were seen for itching, which was common before treatment at 42.5%, but which significantly decreased for the miltefosine (*p* = 0.013) and cryotherapy group (*p* = 0.005), but not for the patients who were not treated (*p* = 0.307).

Agreement between when patient saw the lesion as clear and physicians assessed the lesion to be cured was low with patients rating their response more positively than the physician (Supplementary Table 10), with kappa coefficient − 0.07 at M1, 0.37 at M3 and 0.52 at M6.

## Discussion

This is the first study to comprehensively report both clinical and patient-reported treatment outcomes for CL treatment. We also provide important information on challenges and opportunities of community treatment, which is scarce despite the emphasis placed by the WHO roadmap for NTDs (7) on community-based interventions.

CL is a common morbidity in Ochollo, which is reflected by the low age of the enrolled study patients, and the high number of patients who had previous or active CL cases in their household. These findings are in line with Bugssa et al. (14), who found that about 65% of primary school children had either active lesions or scars due to CL. Regardless of the high CL prevalence, only few patients had used modern treatment. Yet, half of them tried traditional medicine with a local healer in the village. This highlights that patients experience important barriers for seeking healthcare at Arba Minch General Hospital, about 25 km from Ochollo village. There barriers were further explored in a sub-study (manuscript in preparation).

While patients predominantly had lesions on their face, their quality of life was not much affected by CL. Most other studies that used the DLQI in CL patients found much higher impact (27–30), even though lesions were mostly on extremities. Some of the factors that could cause this discrepancy are age, since adults were impacted more, and the high endemicity of CL in the village. Others have shown that lack of knowledge about CL and its transmission can lead to fears related to contamination, causing rejection and isolation in communities (31–33). Researchers have been coming to Ochollo to study CL since the 1970’s (14, 34–37), which probably contributed to good knowledge and accordingly less impact of CL on their quality of life, compared to other sites. This is further highlighted by the fact that 96% of people from Ochollo knew CL is caused by the bite of (sand)flies (36), which is much higher than in other CL-endemic areas in the country (4, 38, 39).

Overall, clinical outcomes of cryotherapy were lower than expected with only a bit more than half of the patients reaching cure at M6. However, an additional fifth of the patients reached substantial improvement. A few other studies done in Ethiopia had higher cure rates; in Silti Health Center, 80.5% of patients treated with cryotherapy were cured at three to 6 months (10). They used a cotton applicator and 3–4 times 10–30 s freeze thaw cycles in weekly sessions up to cure, with an average of 6.4 sessions needed. In ALERT hospital, cure rate with a similar protocol was 60.8% (8), but the majority of patients were treated for more than 13 sessions. A small report from Boru Meda Hospital showed that cryotherapy cure rate was 92.3% after three doses (9), although details on the treatment application and outcome assessment are not described. Generally, we used relatively strict cure criteria, where all lesions had to have complete reepithelization and flattening to be cured at M6, which may not be the case for other studies. The young age of our patients could also play a role in low cure rates, as younger patients have been described to have poorer outcomes in several studies (12, 40–42).

Treatment with cryotherapy came with several challenges and observations. First, due to the very young patient population, Ochollo cryotherapy was applied mostly on small children. Especially the youngest were frightened by the sound of the cryogun, which made it difficult to apply the liquid nitrogen. Difficulties in application of cryotherapy on young patients could also be a cause of lower cure rates. We indeed showed that outcomes were significantly better in those above 5 years. Second, the patients reside in a rural area without access to running water. Although we advised patients to wash their lesion frequently to prevent infections, patients often came back with unclean or infected lesions. Consequently, we provided 2% topical fusidic to keep the wounds clean. Third, despite the aim for weekly cryotherapy application in our protocol, in practice this was almost never possible because lesions were not yet healed from the previous application. Therefore, we recommend application of cryotherapy every other week. Fourth, the number of cryotherapy sessions needed to heal a lesion were highly variable among patients. This is complicating standardization of treatment, which is needed to enable comparison of the effectiveness of cryotherapy with other treatments. Importantly, our results show that extending treatment does not necessarily lead to better outcomes. Therefore, clinicians should carefully assess whether there is sufficient improvement of the lesion after four sessions of cryotherapy before further treatment extension. Lastly, although severe hypopigmentation is often mentioned as a reason to avoid cryotherapy in the face on darker skin, our data showed that while pigmentation issues were common, they were mostly transient with good recovery at M6, similar to findings from Iran (43).

Outcomes of miltefosine treatment in our study were in line with a previous pilot study conducted in Ethiopia in a hospital population (12) where the corrected overall cure rate was 48.7% at M6. In short, patients showed good initial improvement but seemed to be unable to completely clear the infection, as a considerable proportion of patients had lesion worsening at M6 compared to M3. Based on this, we hypothesize that treatment extension or combination treatment could improve cure rates. We observed that adherence of miltefosine was poor, despite extensive efforts by the study team. This may have contributed to lower cure rates, even though our data did not show significantly different outcomes in this group. Closer follow-up by the village health extension worker through house-to-house visits and consulting patients may improve adherence and potentially treatment outcomes. The age limit for systemic treatment was set at age four in our study protocol because we feared patients would be too young to swallow the drug. In practice, this led to unnecessary exclusion of a lot of patients who needed systemic treatment but were below 4 years old. For the youngest patients, we opened the capsules and dissolved the miltefosine in a cup with water and sugar. We therefore recommend future studies to lower inclusion age to 2 years.

Results from our study highlight that the use of oral treatments, − even though deemed the future of leishmaniasis treatment (44) – comes with important challenges for decentralization. Many CL patients live in rural communities and are poorly educated. This requires extensive efforts to sensitize and instruct communities on drug adherence and prevent development of resistance. Directly observed therapy at the health post level, similar to what is done for tuberculosis, could be explored.

Patients who were not treated in our study because they were too young for systemic treatment or because they refused, showed similar cure rates as the treatment groups. Interestingly, especially among the patients who received topical fusidic acid, lesions seemed to improve quickly and had a response rate similar to the cryotherapy and miltefosine groups. It should be taken into account, however, that this non-treatment group only consisted of a few patients and therefore the evidence is anecdotal at best. Further investigation into the (natural) healing of lesions and the added effect of topical antibiotics on CL treatment is warranted as they are cheap, easy to apply and locally available.

Our findings indicate that many patients have an ineffective immune response to CL, shown by the long duration of lesions, high number of patients with new lesions, and a high proportion of patients who have both an active lesion and scar. The persistence of lesions also indicates patients could remain infectious for years and sustain transmission. Patients in Ochollo are presumably frequently exposed to infectious sand fly bites, 3.5% of sand flies were found infected with *Leishmania* in Ochollo (36). However, especially children seem to fail in raising protective immunity to prevent re-infection. Immunological studies should be performed to shed light on this phenomenon, and help determine whether early treatment of patients in a highly endemic setting is useful. In our view, it is more likely that treatment campaigns should be integrated with vector control measures to reduce the patients’ exposure to infectious sand flies.

This is the first time that patient-reported outcomes and scar evaluation were done in conjunction with clinical assessment to give an integrated assessment of treatment outcomes, as recommended previously (13). Our findings show that the impact of CL after treatment measured by the DLQI decreased significantly after treatment, and lesion assessment was significantly better at M6 compared to baseline. Interestingly, patient-reported outcomes were much more positive than clinical outcomes, and did not correlate well with clinical findings. This highlights that it is vital to include patients’ perspectives, as a pure clinical evaluation can underestimate the perceived effect of treatment, which is especially important for skin diseases which mainly have psychosocial impact. Other strengths of this study are good follow-up of patients due to close involvement of field assistants. Future community-based projects should closely involve village leaders and health extension workers in patient management and follow-up in order to increase local ownership and acceptance. Limitations of this study are the relatively low sample size of each treatment group, and potential social desirability bias in the patient-reported outcomes. Since this study provides results from a specific highly endemic locality, certain findings such as the patient population, impact of CL and number of new lesions are not generalizable to other settings. However, most lessons learned can be applied to all CL-endemic communities in Ethiopia.

## Conclusion

Our study is the first to identify the challenges and opportunities of miltefosine and cryotherapy for community treatment of CL. We show that local engagement is crucial for the success of community studies. Application of cryotherapy should be spaced 2 weeks apart and topical antibiotics should be routinely supplied to avoid infection. Pigmentation problems were frequently encountered, but most improved after 6 months. Poor miltefosine adherence highlights that oral outpatient treatments for CL need more stringent follow-up. Cryotherapy and miltefosine are suboptimal in terms of cure-rate, although the majority of patients still experienced great improvement of their lesion. This indicates that patient-reported outcomes are very valuable, especially for skin NTDs. There currently seem to be no other treatments suitable for decentralization readily available. Therefore, integrated interventions aimed to reduce transmission in combination with early diagnosis and treatment should be explored.

## Data availability statement

The datasets presented in this article are not readily available because data will not be made openly accessible due to ethical and privacy concerns. Data can however be made available after approval of a motivated and written request. Requests to access the datasets should be directed to ITM Research Data Access Committee, ITMresearchdataaccess@itg.be.

## Ethics statement

The studies involving humans were approved by Arba Minch University Institutional Research Ethics Review Board Institute of Tropical Medicine Institutional Review Board University Hospital Antwerp Ethics Review Board. The studies were conducted in accordance with the local legislation and institutional requirements. Written informed consent for participation in this study was provided by the participants’ legal guardians/next of kin.

## Author contributions

SvH, MP, JvG, FM, and BM contributed to conception and design of the study. DT, MdK, MT, EK, NG, DD, MM, MS, and RT were involved in field work and data collection. SvH, MP, DT, BM, and TW supervised and coordinated the study. DD and NG did data entry. MbK performed the molecular analyses while MP supervised. SvH organized the database and performed the statistical analysis. SvH and MP wrote the first draft of the manuscript. FM, JvG, and TW provided critical input to the writing of the manuscript. All authors contributed to the article, read, and approved the submitted version.

## References

[1] van Henten S Adriaensen W Fikre H Akuffo H Diro E Hailu A. Cutaneous Leishmaniasis due to Leishmania aethiopica. E Clini Med. (2018) 6:69–81. doi: 10.1016/J.ECLINM.2018.12.009, PMID: 31193672 PMC6537575

[2] Mutinga MJ Odhiambo TR. Cutaneous leishmaniasis in Kenya.2. Studies on vector potential of phlebotomus-pedifer (diptera, phlebotomidae) in Kenya. Insect Sci its Appl. (1986) 7:171–4. doi: 10.1017/S1742758400008924

[3] Pareyn M Kochora A van Rooy L Eligo N vanden Broecke B Girma N. Feeding behavior and activity of Phlebotomus pedifer and potential reservoir hosts of Leishmania aethiopica in southwestern Ethiopia. PLoS Negl. Trop. Dis. (2020) 14:e0007947. doi: 10.1371/journal.pntd.0007947, PMID: 32196501 PMC7112221

[4] Merdekios B Pareyn M Tadesse D Getu S Admassu B Girma N. Detection of cutaneous leishmaniasis foci in south Ethiopia. American J Trop Med Hygiene. (2021) 105:156. doi: 10.4269/ajtmh.20-0708 PMC8274791 33970885

[5] World Health Organization. Global health observatory data repository. World Health Organization (2019).

[6] Alvar J Vélez ID Bern C Herrero M Desjeux P Cano J. Leishmaniasis worldwide and global estimates of its incidence. PLoS One. (2012) 7:e35671. doi: 10.1371/journal.pone.0035671, PMID: 22693548 PMC3365071

[7] World Health Organization. Ending the neglect to attain the sustainable development goals: a road map for neglected tropical diseases 2021–2030. (2020). Available at: https://www.who.int/publications/i/item/9789240010352 [Accessed October 25, 2021]

[8] Leish-mapping team at AHRI in collaboration with WHO-Ethiopia. Proceedings of the *International Consultative Meeting on Cutaneous Leishmaniasis in Ethiopia*. Addis Ababa. (2011).

[9] Seife T Benecha AK Zewdu FT Ayal A Misganaw M. Treatment patterns and Effectivness of anti-Leishmaniasis agents for patients with cutaneous Leishmaniasis at Boru Meda hospital, South Wollo, North East Ethiopia. J Clin Exp Dermatol Res. (2018) 9:1–6. doi: 10.4172/2155-9554.1000450

[10] Negera E Gadisa E Hussein J Engers H Kuru T Gedamu L. Treatment response of cutaneous leishmaniasis due to Leishmania aethiopica to cryotherapy and generic sodium stibogluconate from patients in Silti, Ethiopia. Trans. R. Soc. Trop. Med. Hyg. (2012) 106:496–503. doi: 10.1016/j.trstmh.2012.02.006, PMID: 22503475

[11] Sankaranarayanan R Rajkumar R Esmy PO Fayette JM Shanthakumary S Frappart L. Effectiveness, safety and acceptability of “see and treat” with cryotherapy by nurses in a cervical screening study in India. Br. J. Cancer. (2007) 96:738–43. doi: 10.1038/sj.bjc.6603633, PMID: 17311015 PMC2360066

[12] van Henten S Tesfaye AB Abdela SG Tilahun F Fikre H Buyze J. Miltefosine for the treatment of cutaneous leishmaniasis—a pilot study from Ethiopia. PLoS Negl. Trop. Dis. (2021) 15:e0009460. doi: 10.1371/journal.pntd.0009460, PMID: 34048461 PMC8191986

[13] Erber AC Arana B Bennis I Ben Salah A Boukthir A Castro Noriega MDM. An international qualitative study exploring patients’ experiences of cutaneous leishmaniasis: study set-up and protocol. BMJ Open. (2018) 8:e021372. doi: 10.1136/bmjopen-2017-021372, PMID: 29909372 PMC6009565

[14] Bugssa G. The current status of cutaneous Leishmaniasis and the pattern of lesions in Ochollo primary school students, Ochollo. Southwestern Ethiopia Sci J Clin Med. (2014) 3:111. doi: 10.11648/j.sjcm.20140306.13

[15] Von Elm E Altman DG Egger M Pocock SJ Gøtzsche PC Vandenbroucke JP. The strengthening the reporting of observational studies in epidemiology (STROBE) statement: guidelines for reporting observational studies. Ann. Intern. Med. (2007) 147:573–7. doi: 10.7326/0003-4819-147-8-200710160-00010 17938396

[16] World Health Organization. Control of the leishmaniases. World Health Organization. (2010).

[17] Merdekios B Pareyn M Tadesse D Eligo N Kassa M Jacobs BK. Evaluation of convential and four real-time PCR methods for the detection of Leismania in field-collected samples in Ethiopia. PLoS Negl. Trop. Dis. (2020) 15:e0008903. doi: 10.1371/journal.pntd.0008903, PMID: 33434190 PMC7802924

[18] Steinau M Rajeevan M Unger E. DNA and RNA references for qRT-PCR assays in exfoliated cervical cells. J Mol Diagn. (2006) 8:113–8. doi: 10.2353/JMOLDX.2006.050088, PMID: 16436642 PMC1867570

[19] Federal Ministry of Health E. Guidelines for diagnosis, treatment and prevention of leishmaniasis in Ethiopia. 2nd ed. Ethiopia: Addis Adaba (2013).

[20] Finlay AY Khan GK. Dermatology life quality index (DLQI)—a simple practical measure for routine clinical use. Clin. Exp. Dermatol. (1994) 19:210–6. doi: 10.1111/j.1365-2230.1994.tb01167.x, PMID: 8033378

[21] Finlay AY Sampogna F. What do scores mean? Informed interpretation and clinical judgement are needed. Br. J. Dermatol. (2018) 179:1021–2. doi: 10.1111/BJD.17028, PMID: 30387517

[22] Nedelec B Correa JA Rachelska G Armour A Lasalle L. Quantitative measurement of hypertrophic scar: interrater reliability and concurrent validity. J Burn Care Res. (2008) 29:501–11. doi: 10.1097/BCR.0B013E3181710881, PMID: 18388576

[23] Dorlo TPC Huitema ADR Beijnen JH De Vries PJ. Optimal dosing of miltefosine in children and adults with visceral leishmaniasis. Antimicrob. Agents Chemother. (2012) 56:3864–72. doi: 10.1128/AAC.00292-12, PMID: 22585212 PMC3393397

[24] U.S. Department of Health and Human Services. Common terminology criteria for adverse events (CTCAE).V.5.0. Cancer Ther Eval Progr. (2017). 155. Available at: http://upen.terengganu.gov.my/index.php/2017

[25] Harris PA Taylor R Thielke R Payne J Gonzalez N Conde JG. Research electronic data capture (REDCap)—a metadata-driven methodology and workflow process for providing translational research informatics support. J. Biomed. Inform. (2009) 42:377–81. doi: 10.1016/J.JBI.2008.08.010, PMID: 18929686 PMC2700030

[26] Team RC. R: A language and environment for statistical computing. Austria: R Found Stat Comput Vienna (2023).

[27] Vares B Mohseni M Heshmatkhah A Farjzadeh S Safizadeh H Shamsi-Meymandi S. Quality of life in patients with cutaneous leishmaniasis. Arch Iran Med. (2013) 16:474–7. 23906253

[28] Ahmed N Naeem A Zahid B Tahir M Bashir U Kausar S. Effect of cutaneous Leishmaniasis on quality of life of patients, a multicentric study in tertiary care hospitals in Pakistan using DLQI. Int J Clin Experim Med Sci. (2021) 7:103. doi: 10.11648/J.IJCEMS.20210704.16

[29] de Castro Toledo AC da Silva RE Carmo RF Amaral TA Profeta Luz Z Lia M. Assessment of the quality of life of patients with cutaneous leishmaniasis in belo Horizonte, Brazil, 2009-2010. A pilot study. Trans. R. Soc. Trop. Med. Hyg. (2013) 107:335–6. doi: 10.1093/trstmh/trt021, PMID: 23474473

[30] Peleva E Walker SL. Cutaneous leishmaniasis and health-related quality of life in returning travellers to the UK. J Travel Med. (2020) 27:1–2. doi: 10.1093/jtm/taaa188, PMID: 33145597 PMC7883819

[31] Chahed MK Bellali H Ben Jemaa S Bellaj T. Psychological and psychosocial consequences of zoonotic cutaneous Leishmaniasis among women in Tunisia: preliminary findings from an exploratory study. PLoS Negl. Trop. Dis. (2016) 10:e0005090. doi: 10.1371/JOURNAL.PNTD.0005090, PMID: 27788184 PMC5082956

[32] Reyburn H Koggel M Sharifi AS. Social and psychological consequences of cutaneous leishmaniasis in Kabul Afghanistan. United States: University of Arizona Libraries (2000).

[33] Ramdas S. Perceptions and treatment of cutaneous leishmaniasis in Suriname: a medical-anthropological perspective. Netherlands: University of Amsterdam (2015).

[34] Ashford RW Bray MA Hutchinson MP Bray RS. The epidemiology of cutaneous leishmaniasis in Ethiopia. Trans. R. Soc. Trop. Med. Hyg. (1973) 67:568–601. doi: 10.1080/00034983.1973.11811669 4150462

[35] Mengistu G Laskay T Gemetchu T Humber D Ersamo M Evans D. Cutaneous leishmaniasis in South-Western Ethiopia: Ocholo revisited. Trans. R. Soc. Trop. Med. Hyg. (1992) 86:149–53. doi: 10.1016/0035-9203(92)90546-O, PMID: 1440773

[36] Pareyn M van den Bosch E Girma N van Houtte N van Dongen S van der Auwera G. Ecology and seasonality of sandflies and potential reservoirs of cutaneous leishmaniasis in Ochollo, a hotspot in southern Ethiopia. PLoS Negl. Trop. Dis. (2019) 13:e0007667. doi: 10.1371/journal.pntd.0007667, PMID: 31425506 PMC6715250

[37] Kebede N Worku A Ali A Animut A Negash Y Gebreyes WA. Community knowledge, attitude and practice towards cutaneous leishmaniasis endemic area Ochello, Gamo Gofa zone, South Ethiopia. Asian Pac. J. Trop. Biomed. (2016) 6:562–7. doi: 10.1016/J.APJTB.2016.01.018

[38] Tamiru HF Mashalla YJ Mohammed R Tshweneagae GT. Cutaneous leishmaniasis a neglected tropical disease: community knowledge, attitude and practices in an endemic area. Northwest Ethiopia BMC Infect Dis. (2019) 19:1–10. doi: 10.1186/s12879-019-4506-1, PMID: 31619180 PMC6796443

[39] Tesfay K Mardu F Berhe B Negash H Legese H Adhanom G. Household knowledge, practice and treatment seeking behaviors towards cutaneous leishmaniasis in the endemic rural communities of Ganta- afeshum district, Tigrai, northern Ethiopia, 2019: a cross-sectional study. Trop Dis Travel Med Vaccines. (2021) 7:1–10. doi: 10.1186/S40794-021-00144-4/TABLES/4 34130733 PMC8204582

[40] Layegh P Rahsepar S Rahsepar AA. Systemic Meglumine Antimoniate in acute cutaneous Leishmaniasis: children versus adults. Am J Trop Med Hyg. (2011) 84:539–42. doi: 10.4269/AJTMH.2011.10-0002, PMID: 21460006 PMC3062445

[41] Llanos-Cuentas A Tulliano G Araujo-Castillo R Miranda-Verastegui C Santamaria-Castrellon G Ramirez L. Clinical and parasite species risk factors for pentavalent antimonial treatment failure in cutaneous Leishmaniasis in Peru. Clin. Infect. Dis. (2008) 46:223–31. doi: 10.1086/524042, PMID: 18171254

[42] Castro M del M Cossio A Velasco C Osorio L. Risk factors for therapeutic failure to meglumine antimoniate and miltefosine in adults and children with cutaneous leishmaniasis in Colombia: a cohort study. PLoS Negl. Trop. Dis. (2017) 11:e0005515. doi: 10.1371/journal.pntd.0005515, PMID: 28379954 PMC5393627

[43] Layegh P Pezeshkpoor F Soruri AH Naviafar P Moghiman T. Efficacy of cryotherapy versus intralesional meglumine antimoniate (glucantime) for treatment of cutaneous leishmaniasis in children. Am J Trop Med Hyg. (2009) 80:172–5. doi: 10.4269/ajtmh.2009.80.172, PMID: 19190206

[44] Drugs for Neglected Diseases Initiative. (2023). Target product profile for cutaneous leishmaniasis | DNDi. Available at: https://dndi.org/diseases/cutaneous-leishmaniasis/target-product-profile/ [Accessed February 21, 2023]

