# Supplementary material for: Community-based treatment of cutaneous leishmaniasis using cryotherapy and miltefosine in Southwest Ethiopia: the way forward?
Source: Front Med (Lausanne). 2023 Oct 10;10:1196063. doi: 10.3389/fmed.2023.1196063 (PMC10598589; doi:10.3389/fmed.2023.1196063)

Supplementary Material

Community-based treatment of cutaneous leishmaniasis using cryotherapy and miltefosine in Southwest Ethiopia: the way forward?

Saskia van Henten^1^†, Myrthe Pareyn†^1^*, Dagimawie Tadesse^2^, Mekidim Kassa^2^, Mehret Techane^2^, Eyerusalem Kinfe^2^, Nigatu Girma^2^, Degnet Demeke^2^, Mebratu Mesay^2^, Mekibib Kassa^3^, Rodas Temesgen^2^, Misgun Shewangizaw^2^, Fekadu Massebo^2^, Johan van Griensven^1^, Teklu Wegayehu^2^, Behailu Merdekios^2^

*** Correspondence:** Myrthe Pareyn: [myrthepareyn@itg.be](mailto:myrthepareyn@itg.be)

# Supplementary Tables

**Supplementary Table 1. Dermatological life quality index categories before and after treatment**

| **Category** | **Baseline Total (%^a^)**  **N=64^b^ (59.8)** | **M1 (%)**  **N=49^c^ (56.3)** | **M3 (%)**  **N=50^d^ (57.5)** | **M6 (%)**  **N=63^e^ (63.0)** |
| --- | --- | --- | --- | --- |
|  | **n (%)** | **n (%)** | **n (%)** | **n (%)** |
| No effect | 37 (57.8) | 43 (87.8) | 46 (92.0) | 60 (95.2) |
| Small effect | 22 (34.4 ) | 4 (8.2) | 4 (8.0) | 3 (4.8) |
| Moderate effect | 3 (4.7) | 1 (2.0) | 0 (0) | 0 (0) |
| Very large effect | 0 (0) | 1 (2.0) | 0 (0) | 0 (0) |
| Extremely large effect | 2 (3.1) | 0 (0) | 0 (0) | 0 (0) |

^a^Percentages are calculated taking all patients who came for the respective visit as the denominator. ^b^Results not available for 32 patients below the age of 4, 2 adults for whom results were invalid, 8 children for whom results were invalid, and for 1 child results were missed. ^c^Results not available for 20 patients who missed M1, 17 below the age of 4, 1 adult for whom results were invalid, 19 children for whom results were invalid, and 1 adult for whom results were missed. ^d^Results not available for 19 patients who missed M3, 17 below the age of 4, 2 adults for whom results were invalid, 19 children for whom results were invalid. ^e^Results not available for 7 patients who missed M6, 28 below the age of 4, 1 adult for whom results were invalid, 8 children for whom results were invalid

**Supplementary Table 2. Side effects of cryotherapy treatment**

| **Side-effect** | **N = 48**  **n (%^a^)** | **Severity I**  **n (%^b^)** | | **Severity II n (%^b^)** | | **Week 1**  **n (%^c^)** | | | **Week2**  **n (%^c^)** | **Week3**  **n (%^c^)** | | **Week 4**  **n (%^c^)** |
| --- | --- | --- | --- | --- | --- | --- | --- | --- | --- | --- | --- | --- |
| Pain | 5 (10.4) | 3 (60.0) | | 2 (40.0) | | 5 (100) | | | 0 (0) | 0 (0) | | 0 (0) |
| Redness | 9 (18.8) | 7 (77.8) | | 2 (22.2) | | 9 (100) | | | 1 (11.1) | 1 (11.1) | | 0 (0) |
| Swelling | 34 (70.8) | 31 (91.2) | | 3 (8.8) | | 29 (85.3) | | | 3 (8.8) | 4 (11.8) | | 5 (14.7) |
| Infection | 28 (58.3) | 21 (75.0) | | 7 (25.0) | | 23 (82.1) | | | 5 (17.9) | 1 (3.6) | | 0 (0) |
| Blistering | 37 (77.1) | 33 (89.2) | | 4 (10.8) | | 35 (94.6) | | | 3 (8.1( | 5 (13.5) | | 5 (13.5) |
| Pigmentation changes | 16^d^ (33.3) | 12 (75.0) | | 2 (12.5) | | 1 (6.3) | | | 5 (31.3) | 7 (43.8) | | 8 (50.0) |
| Any side-effect | 43 (89.6%) | |  | |  | |  |  | | |  |  |

^a^For the number and proportion of each symptom, 48 was used as denominator

^b^For these proportions, the total of number who experienced a specific symptom was taken as denominator. The highest severity was counted for each patient

^c^For these proportions, the total of number who experienced a specific symptom was taken as denominator. Each week the patient had the symptom was counted separately.

^d^Severity of pigmentation changes was not filled twice

| **Side-effect** | | **N =42**  **n (%^a^)** | **Grade I**  **n (%^b^)** | **Grade II**  **n (%^b^)** | | **Grade III**  **n (%^b^)** | | **Week 1**  **n (%^c^)** | **Week 2**  **n (%^c^)** | **Week3**  **n (%^c^)** | **Week 4**  **n (%^c^)** |
| --- | --- | --- | --- | --- | --- | --- | --- | --- | --- | --- | --- |
| Vomiting | | 20 (47.6) | 16 (80.0) | 4 (20.0) | | 0 (0) | | 13 (65.0) | 11 (55.0) | 6 (30.0) | 3 (15.0) |
| Abdominal pain | | 14 (33.3) | 12 (85.7) | 1 (7.1) | | 1^d^ (7.1) | | 4 (28.6) | 7 (50.0) | 6 (42.9) | 3 (21.4) |
| Nausea | | 14 (33.3) | 11 (78.6) | 2 (14.3) | | 0 (0) | | 5 (35.7) | 5 (35.7) | 5 (35.7) | 2 (14.3) |
| Diarrhea | | 9 (21.4) | 8 (88.9) | 0 (0) | | 1^d^ (11.1) | | 4 (44.4) | 3 (33.3) | 3 (33.3) | 3 (33.3) |
| Headache | | 6 (14.3) | 4 (66.7) | 1 (16.7) | | 1^d^ (16.7) | | 1 (16.7) | 2 (33.3) | 2 (33.3) | 3 (33.3) |
| Loss of appetite | | 3 (7.1) | 1 (33.3) | 2 (66.7) | | 0 (0) | | 1 (33.3) | 1 (33.3) | 1 (33.3) | 2 (66.6) |
| Weakness | | 1 (2.4) | 0 (0) | 0 (0) | | 1^d^ (100) | | 1 (100) | 0 (0) | 0 (0) | 1 (100) |
| Fever | | 1 (2.4) | 0 (0) | 0 (0) | | 0 (0) | | 0 (0) | 1 (100) | 1 (100) | 0 (0) |
| Any side-effect | 32 (76.2%) | | | |  | |  |  |  |  |  |

**Supplementary Table 3. Side-effects experienced in patients receiving miltefosine**

^a^For the number and proportion of each symptom, 42 was used as denominator. ^b^For these proportions, the total of number who experienced a specific symptom was taken as denominator. The highest severity was counted for each patient

^c^For these proportions, the total of number who experienced a specific symptom was taken as denominator. Each week the patient had the symptom was counted separately. ^d^One patient had bloody diarrhea with headache, weakness and abdominal comfort which together kept her from getting out of the house. As she responded well to systemic antibiotics it was interpreted as bacterial diarrhea, and likely not related to miltefosine, although it cannot be ruled out completely

**Supplementary Table 4. Treatment outcomes for cryotherapy for the index lesion only**

|  | **Month 1** | | | **Month 3** | | | **Month 6** | |
| --- | --- | --- | --- | --- | --- | --- | --- | --- |
|  | | **N=47 (97.9)** | | | **N=48 (100)** | | **N=46 (95.8)** | |
|  | | **n (%)** | **95% CI** | | **n (%)** | **95% CI** | **n (%)** | **95% CI** |
| Cure | | 12 (25.5) | 12.8 - 40.6 | | 23 (47.9) | 35.4 - 63.8 | 24 (52.2) | 39.1 - 67.6 |
| Substantial improvement | | 26 (55.3) | 42.6 - 70.3 | | 15 (31.3) | 18.8 - 47.1 | 10 (21.7) | 8.7 - 37.2 |
| Minor improvement | | 6 (12.8) | 0 - 27.8 | | 4 (8.3) | 0 - 24.2 | 2 (4.3) | 0 - 19.8 |
| No improvement | | 2 (4.3) | 0 - 19.3 | | 0 (0) | 0 - 15.9 | 1 (2.2) | 0 - 17.6 |
| Worsening | | 1 (0.2) | 0 - 17.1 | | 6 (12.5) | 0 - 28.4 | 9 (19.6) | 6.5 - 35.0 |

**Supplementary Table 5. Treatment outcomes for cryotherapy by age category at Month 6**

|  | **Age <5 years**  **N=24** | | | **Age >5 years**  **N=24^a^** | | *P = 0.036^b^* |
| --- | --- | --- | --- | --- | --- | --- |
|  | | **n (%)** | **95% CI** | **n (%)** | **95% CI** |  |
| Cure | | 12 (50.0) | 33.3 - 72.3 | 12 (54.5) | 36.4 - 76.2 |  |
| Substantial improvement | | 2 (8.3) | 0 - 30.7 | 7 (31.8) | 13.6 - 53.4 |  |
| Minor improvement | | 2 (8.3) | 0 - 30.7 | 0 (0) | 0 - 21.6 |  |
| No improvement | | 0 (0) | 0 - 22.3 | 1 (4.5) | 0 - 26.2 |  |
| Worsening | | 8 (33.3) | 16.7 - 55.7 | 2 (9.1) | 0 - 30.7 |  |

^a^Two outcomes of patients >5 are missing

^b^Fisher-exact test

**Supplementary Table 6. Treatment outcome for miltefosine for the index lesion only**

|  | **Month 1**  **N=39 (92.9%)** | | **Month 3**  **N=38 (90.5%)** | | **Month 6**  **N=39 (92.9%)** | |
| --- | --- | --- | --- | --- | --- | --- |
|  | **n (%)** | **95% CI** | **n (%)** | **95% CI** | **n (%)** | **95% CI** |
| Cure | 7 (17.9) | 2.6 - 34.1 | 17 (44.7) | 31.6 - 63.1 | 20 (51.3) | 38.5 - 68.8 |
| Substantial improvement | 19 (48.7) | 33.3 - 64.9 | 16 (42.1) | 28.9 - 60.5 | 10 (25.6) | 12.8 - 43.1 |
| Minor improvement | 13 (33.3) | 17.9 - 49.5 | 3 (7.9) | 0 - 26.3 | 3 (7.7) | 0 - 25.2 |
| No improvement | 0 (0) | 0 - 16.1 | 0 (0) | 0 - 18.4 | 2 (5.1) | 0 - 22.6 |
| Worsening | 0 (0) | 0 - 16.1 | 2 (5.3) | 0 - 23.7 | 4 (10.3) | 0 - 27.7 |

**Supplementary Table 6. Treatment outcome for miltefosine for the index lesion only**

|  | **Month 1**  **N=39 (92.9%)** | | **Month 3**  **N=38 (90.5%)** | | **Month 6**  **N=39 (92.9%)** | |
| --- | --- | --- | --- | --- | --- | --- |
|  | **n (%)** | **95% CI** | **n (%)** | **95% CI** | **n (%)** | **95% CI** |
| Cure | 7 (17.9) | 2.6 - 34.1 | 17 (44.7) | 31.6 - 63.1 | 20 (51.3) | 38.5 - 68.8 |
| Substantial improvement | 19 (48.7) | 33.3 - 64.9 | 16 (42.1) | 28.9 - 60.5 | 10 (25.6) | 12.8 - 43.1 |
| Minor improvement | 13 (33.3) | 17.9 - 49.5 | 3 (7.9) | 0 - 26.3 | 3 (7.7) | 0 - 25.2 |
| No improvement | 0 (0) | 0 - 16.1 | 0 (0) | 0 - 18.4 | 2 (5.1) | 0 - 22.6 |
| Worsening | 0 (0) | 0 - 16.1 | 2 (5.3) | 0 - 23.7 | 4 (10.3) | 0 - 27.7 |

**Supplementary Table 7. Treatment outcomes at month 6 for miltefosine treatment by Allometric dosing or not**

|  | **Allometric**  **N=25^a^** | | **Non-allometric**  **N=16b** | | *P = 0.521^c^* | |
| --- | --- | --- | --- | --- | --- | --- |
|  | **N (%)** | **95% CI** | **N (%)** | **95% CI** | |  |
| Cure | 8 (34.8) | 17.4 - 58.0 | 7 (46.7) | 26.7 - 75.3 | |  |
| Substantial improvement | 7 (30.4) | 13.0 - 53.7 | 4 (26.7) | 6.7 - 55.3 | |  |
| Minor improvement | 3 (13.0) | 0 - 36.3 | 0 (0) | 0 - 28.6 | |  |
| No improvement | 0 (0) | 0 - 23.2 | 1 (6.7) | 0 - 35.3 | |  |
| Worsening | 5 (21.7) | 4.3 - 45.0 | 3 (20.0) | 0 - 48.6 | |  |

^a^ Month 6 results are missing for 2 patients who received allometric dosing

^b^ Month 6 results are missing for 1 patient who received only weight-based dosing

^c^Fisher-exact test

**Supplementary Table 8. Modified Vancouver scar scale for patients with scars at outcome visits**

|  |  | **M1 overall** | **M3 overall** | **M6 overall** |
| --- | --- | --- | --- | --- |
|  | **n/N^a^ (%)** | **72/87 (82.8)** | **66/86 (76.7)** | **79/100 (79.0)** |
| **Overall mVSS score, median, (IQR)** | | 1 (1.0 - 4.0) | 1.0 (1.0 - 3.0) | 1 (1.0 - 4.0) |
| Pliability | Normal | 57 (79.2) | 43 (65.2) | 44 (55.7) |
|  | Supple | 4 (5.6) | 10 (15.2) | 21 (26.6) |
|  | Yielding | 0 (0) | 1 (1.5) | 6 (7.6) |
|  | Firm | 10 (13.9) | 12 (18.2) | 8 (10.1) |
|  | Adherent | 1 (1.39) | 0 (0) | 0 (0) |
| Height | Normal | 57 (79.2) | 43 (65.2) | 44 (55.7) |
|  | 1-2 mm | 4 (5.6) | 10 (15.2) | 21 (26.6) |
|  | 3-4 mm | 0 (0) | 1 (1.5) | 6 (7.6) |
|  | 5-6 mm | 10 (13.9) | 12 (18.2) | 8 (10.1) |
|  | >6 mm | 1 (1.4) | 0 (0) | 0 (0) |
| Vascularity | Normal | 68 (94.4) | 0 (0) | 75 (94.9) |
|  | Pink | 4 (5.6) | 0 (0) | 2 (2.5) |
|  | Red | 0 (0) | 0 (0) | 2 (2.5) |
|  | Purple | 0 (0) | 0 (0) | 0 (0) |
| Pigmentation (hypo/ hyperpigmented) | Normal | 5 (6.9) | 4 (6.1) | 11 (13.9) |
|  | Slightly | 60 (83.3) | 49 (74.2) | 52 (65.8) |
|  | Moderately | 2 (2.8) | 8 (13.6) | 14 (17.7) |
|  | Severely | 5 (6.9) | 4 (6.1) | 2 (2.5) |

IQR: interquartile range, M1: month 1, M3: month 3, M6: Month 6, mVSS: modified Vancouver scar scale. **^a^**n/N indicates number of patients who had a scar amongst those who came for the visit

**Supplementary Table 9. Pain and itching before and after treatment**

|  | **Pain** | | |  | **Itching** | | |  |
| --- | --- | --- | --- | --- | --- | --- | --- | --- |
|  | **D0** | **M6** | P-value^a^ | | | **D0** | **M6** | P-value^a^ |
|  | N (%) | N (%) |  | | | N (%) | N (%) |  |
| Total | 40/106 (37.7) | 3/100 (3.0) | <0.001 | | | 45/106 (42.5) | 7/100 (7.0) | <0.001 |
| Cryotherapy | 13/48 (27.1) | 0/46 (0) | <0.001 | | | 19/48 (39.6) | 4/46 (8.7) | <0.001 |
| Miltefosine | 15/42 (35.7) | 2/39 (5.1) | 0.001 | | | 17/42 (40.5) | 3/39 (7.7) | 0.001 |
| No treatment | 12/16 (75.0) | 1/15 (6.7) | <0.001 | | | 9/16 (56.3) | 0/15 (0) | 0.015 |

D0: day 0, M6: month 6. ^a^Chi-square test

**Supplementary Table 10. Agreement between patient-reported and clinical outcome**

|  | **Cure** | **Not cure** | Kappa-coefficient |
| --- | --- | --- | --- |
| **Month 1 assessment** |  |  | -0.07 |
| Clear | 2 | 17 |  |
| Not Clear | 11 | 54 |  |
| **Month 3 assessment** |  |  | 0.37 |
| Clear | 31 | 22 |  |
| Not Clear | 5 | 20 |  |
| **Month 6 assessment** | | | 0.52 |
| Clear | 46 | 20 |  |
| Not Clear | 2 | 25 |  |
|  |  |  |  |

# Supplementary Figures

**Supplementary Figure 1.** Flowchart of screened and included patients.


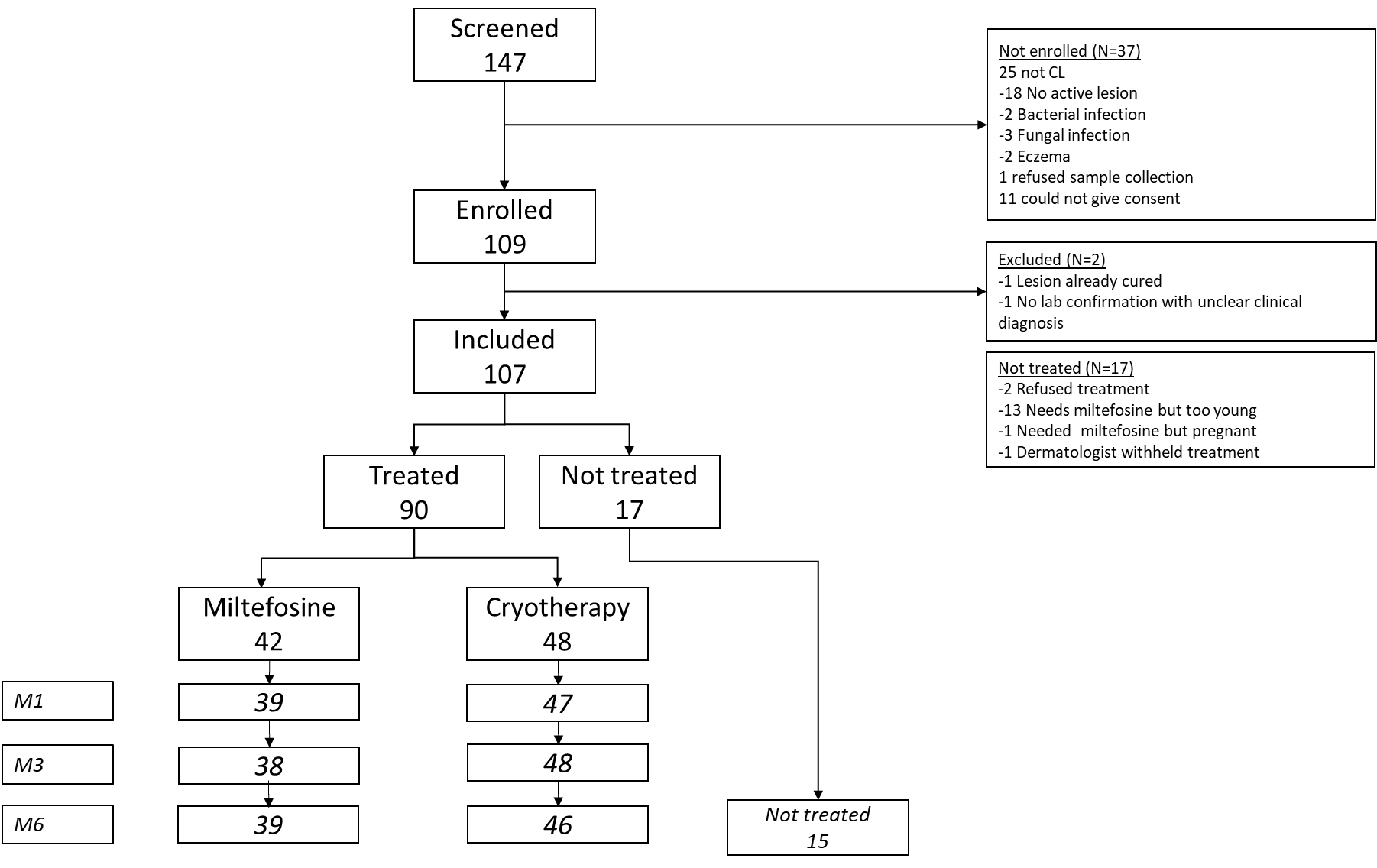

Supplement: Supplementary file 2 [file Data_Sheet_2.docx]
